# Synthetic Methods and Pharmacological Potentials of Triazolothiadiazines: A Review

**DOI:** 10.3390/molecules29061326

**Published:** 2024-03-16

**Authors:** Mohamed S. Mostafa, Ibrahim Ali M. Radini, Naglaa M. Abd El-Rahman, Rizk E. Khidre

**Affiliations:** 1 Department of Physical Sciences, Chemistry Division, College of Science, Jazan University, P.O. Box 114, Jazan 45142, Saudi Arabia; iradini@jazanu.edu.sa; 2 Chemical Industry Research Institute, National Research Centre, Dokki, Giza P.O. Box 12622, Egypt; naglaa_r@yahoo.com

**Keywords:** triazolo[3,4-*b*]thiadiazines, triazolo[5,1-*b*]thiadiazines, triazolo[4,3-*c*]thiadiazines triazol, thiadiazine, thiocarbohydrazide, biological activity

## Abstract

This review article examines the synthetic pathways for triazolothiadiazine derivatives, such as triazolo[3,4-*b*]thiadiazines, triazolo[5,1-*b*]thiadiazines, and triazolo[4,3-*c*]thiadiazines, originating from triazole derivatives, thiadiazine derivatives, or thiocarbohydrazide. The triazolothiadiazine derivatives exhibit several biological actions, including antibacterial, anticancer, antiviral, antiproliferative, analgesic, anti-inflammatory, and antioxidant properties. The review article aims to assist researchers in creating new biologically active compounds for designing target-oriented triazolothiadiazine-based medicines to treat multifunctional disorders.

## 1. Introduction

**Figure 1:**
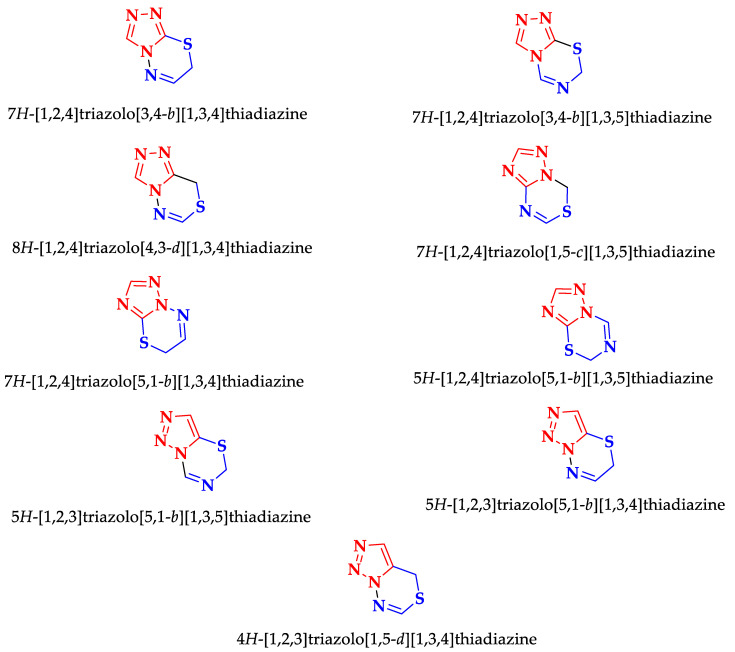
Isomeric forms of triazolothiadiazine.

In recent years, nitrogen- and sulfur-containing scaffolds have become prominent in health, agriculture, and industry due to their extensive applications [1]. One nitrogen- and sulfur-containing heterocycle is triazolothiadiazine. This is a bicyclic molecule of nine members, consisting of four carbon atoms, four nitrogen atoms, and one sulfur atom. This heterocycle is generated by the combination of two active components, a five-membered triazole and a six-membered thiadiazine. As shown in Figure 1, triazolothiadiazine has nine types of isomers.

**Figure 2:**
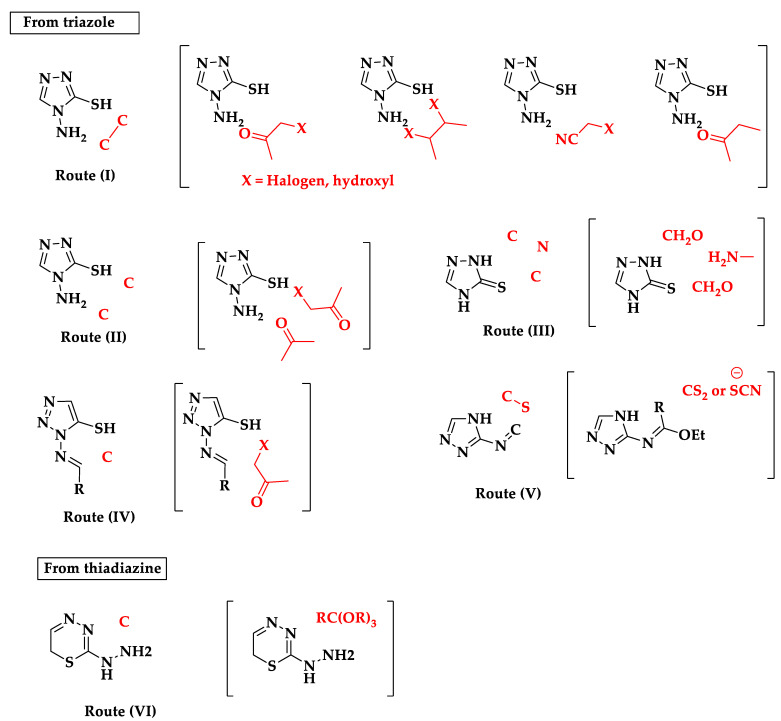
Synthetic methodologies involving triazolothiadiazines.

Triazolothiadiazine derivatives have a wide range of therapeutic activities such as antimicrobial [2,3], tubulin inhibition [4], anticancer [5,6,7,8], anti-candida [9], and antiviral activity against highly pathogenic avian influenza (HPAI H_5_N_1_) [10], antiviral [11], antiproliferative [12], aromatase inhibition [13], analgesic, anti-inflammatory, and antioxidant effects [14,15]. Figure 2 depicts the retrosynthetic analysis used to create the triazolothiadiazine derivatives, involving six main routes: **I**, **II**, **III**, **IV**, **V**, and **VI**. Route **I** consists of the reaction between 4-amino-5-mercaptotriazole and several α-halocarbonyl compounds. Route **II** entails alkylating the thiol group of triazole followed by cyclization using a carbonyl chemical. A one-pot Mannich reaction of 1,2,4-triazole-5-thiones, formaldehyde, and primary amines is present in Route **III**. Route **IV** consists of the reaction between triazole arylidene and *α*-halocarbonyl molecules. Route **V** entails the reaction of triazol-3-amine with ortho esters, followed by the reaction of the generated carboximidate with either carbon disulfide or sodium thiocyanate. Route **VI** consists of the reaction between 2-hydrazinyl-1,3,4-thiadiazine and *α*-halocarbonyl compounds. Aggarwal, R. et al. [16] conducted a review on the synthetic and medicinal aspects of 1,2,4-triazolo[3,4-*b*]1,3,4-thiadiazines. This study discusses the synthetic methods and biological activity of many triazolothiadiazine derivatives, specifically triazolo[3,4-*b*]thiadiazines, triazolo[5,1-*b*]thiadiazines, triazolo[4,3-*c*]thiadiazines, and triazolo[1,5-*c*]thiadiazines building upon our previous research [17,18,19,20,21,22].

## 2. Synthesis of Triazolo[3,4-*b*]thiadiazines

### 2.1. From 4-Amino-5-substituted-4H-1,2,4-triazole-3-thiols

**Scheme 1:**
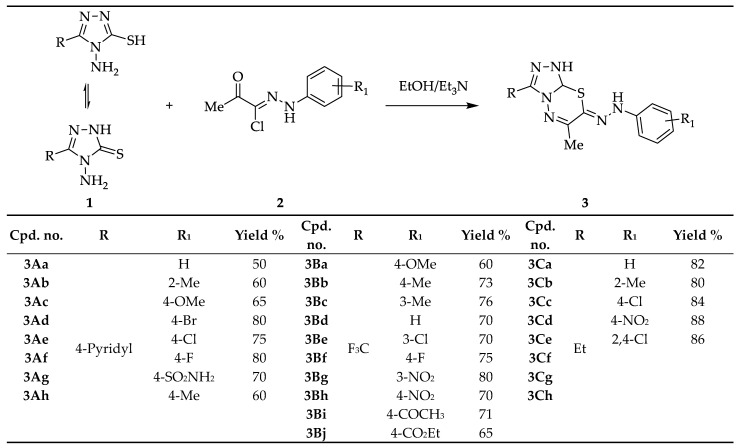
Synthesis of -[1,2,4]triazolo[3,4-*b*][1,3,4]thiadiazines **3A** [6], **3B** [9], and **3C** [23].

The reaction of 4-amino-5-substituted-4*H*-1,2,4-triazole-3-thiols **1** with hydrazonoyl chlorides **2** in refluxing ethanol or dioxane with Et_3_N afforded (7Z)-7-[2-(aryl)hydrazinylidene]-6-methyl-7*H*-[1,2,4]triazolo[3,4-*b*][1,3,4]thiadiazines **3** [6,9,10,11,12,13,14,15,16,17,18,19,20,21,22,23]. The produced compounds underwent in vitro testing against 11 candida species and were compared to the conventional medication ketoconazole. Triazolothiadiazines **3Aa**, **3Ac**, **3Ae**, and **3Ag** showed the highest potency against candida species. The potential cytotoxicity of all the produced compounds was also examined on a noncancer cell line (MCF-12) utilizing the WST-1 test. The compounds **3Aa, 3Ad**, and **3Ah** exhibited an equivalent IC_50_ value to the standard medicine ketoconazole against the noncancer cell line MCF-12 (IC_50_ ≥ 1.0 × 10^5^ µg/mL) [9]. Furthermore, the synthesized compounds underwent a screening process to evaluate their effectiveness in inhibiting cancer growth. Compounds **3Ba** and **3Bi** showed the most significant effects against HEPG-2, indicating promising findings [6] (Scheme 1).

**Scheme 2:**
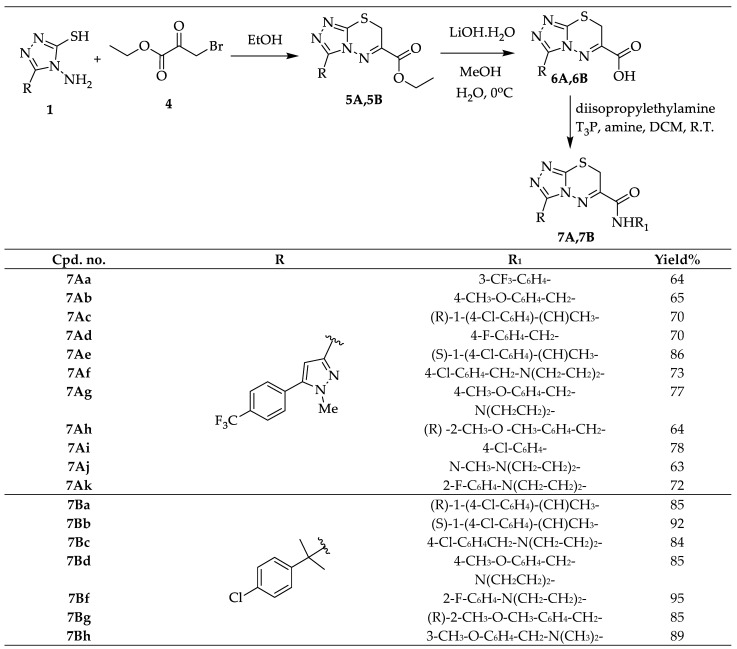
Synthesis of [1,2,4]triazolo[3,4-*b*][1,3,4]thiadiazines **7A** and **7B**.

4-Amino-5-substituted-4*H*-1,2,4-triazole-3-thiols **1** were treated with ethyl 3-bromo-2-oxopropanoate **4** in ethanol at reflux to afford ethyl triazolothiadiazine-6-carboxylate **5A**,**B**. Hydrolyzing the latter esters using lithium hydroxide in MeOH/H_2_O gave the corresponding carboxylic acids **6A**,**B**. The amides **7A**,**B** were synthsized, in good yields, by reacting **6A**,**B** with substituted amines and T3P^®^ in DCM with *N,N*-diisopropylethylamine (Scheme 2). The synthesized compounds underwent screening to evaluate their cytotoxic, anti-inflammatory, and analgesic properties. The compound **7Ag**, (R = 4-(methoxybenzyl)piperazine) has the greatest potential IC_50_ = 9.1 µg/mL in MCF-7 cells. Compounds **7Af**, **7Ag**, and **7Ak** had remarkable anti-inflammatory effects at a dosage of 50 mg/kg, which were nearly equivalent to those of the conventional medication. In addition, the latter compounds exhibited greater and more consistent analgesic effectiveness at doses of 100 and 200 mg/kg/po, while posing a lower risk of causing ulcers [24].

**Scheme 3:**
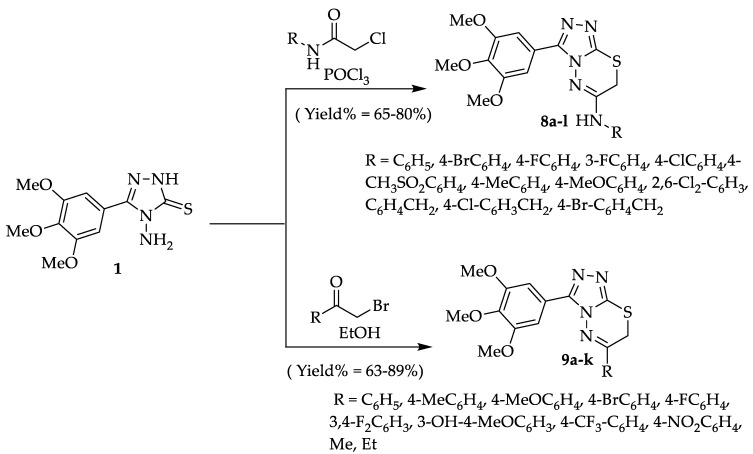
Synthesis of -[1,2,4]triazolo[3,4-*b*][1,3,4]thiadiazines **8 and 9**.

Reaction of 4-amino-3-(3,4,5-trimethoxyphenyl)-1*H*-1,2,4-triazole-5(4*H*)-thione **1** with 2-chloroacetamides in phosphorous oxychloride under refluxing gave *N*-aryl-1,8a-dihydro-7*H*-[1,2,4]triazolo[3,4-*b*][1,3,4]thiadiazin-6-amines **8**, while reaction between **1** and α-bromoketones in anhydrous EtOH under reflux afforded 6-aryl-1,8a-dihydro-7*H*-[1,2,4]triazolo[3,4-*b*][1,3,4]thiadiazines **9** (Scheme 3) [25]. Among synthesized compounds, the most potent compound was **9b** (R = 4-Me-Ph). High selectivity over normal human embryonic kidney HEK-293 cells was observed, with an IC_50_ value over 64.5 μg/mL. Further investigation demonstrated that **9b** prevented tubulin polymerization and disrupted the A549 microtubule network. **9b** changed p-cdc2 and cyclin B1 levels to terminate the G2/M cell cycle. It increased cleaved PARP and caspase-3 and decreased Bcl-2, killing cells. No harm was observed in a xenograft mice model where **9b** inhibited A549 lung cancer growth, demonstrating its potential as a cancer therapy [25].

**Scheme 4:**
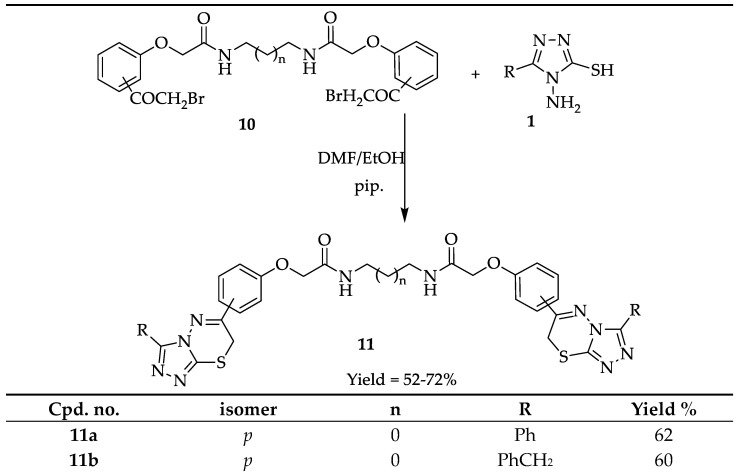

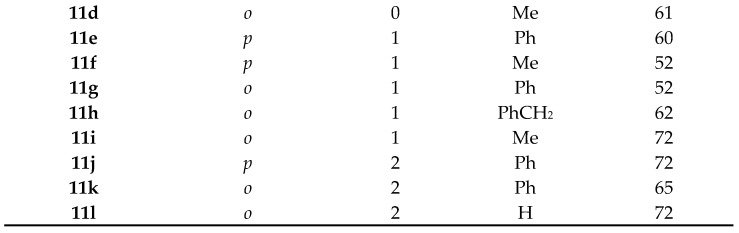
Synthesis of bis(1,2,4-triazolo[3,4-*b*][1,3,4]thiadiazine) derivatives **11**.

Reaction of bis(2-bromoacetyl)phenoxy)acetamides **10** with compound **1** in DMF/ethanol (4:1) at reflux with piperidine furnished bis(1,2,4-triazolo[3,4-*b*][1,3,4]thiadiazine) derivatives **11a–l,** in good yields (Scheme 4). Compounds **11a–l** were tested for cytotoxicity and apoptosis via PARP-1 and EGFR. Three compounds, **11d**, **11i**, and **11l**, outperformed Erlotinib in MDA-MB-231 cytotoxicity. Compound **11i** increased P53, Bax, caspase-3, caspase-8, and caspase-9 gene levels and decreased Bcl2 levels in MDA-MB-231 cells, causing 38-fold apoptosis compared to the control. Compound **11i** showed promising dual enzyme inhibition of PARP-1 (IC_50_ = 0.88 μg/mL) and EGFR (IC_50_ = 64.65 μM) compared to Erlotinib (IC_50_ = 51.6 μg/mL). Thus, compound **11i** may fight breast cancer [7].

**Scheme 5:**
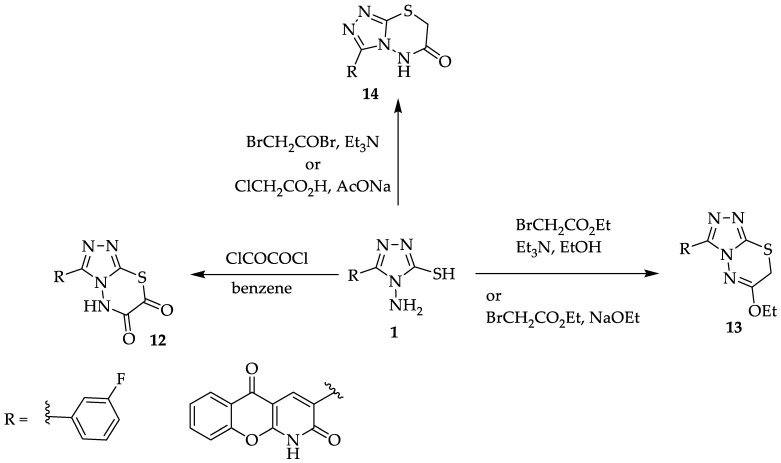
Synthesis triazolothiadiazine derivatives **12–14**.

Compound **1** is treated with oxalyl chloride in refluxing benzene for 6 h to produce 78% [1,2,4]triazolo[3,4-*b*][1,3,4]thiadiazine-6,7-dione **12**. Microwave irradiation (MWI) for 2 min increased the isolated yield to 90%. Moreover, Compound **1** was carboxymethylated with ethyl bromoacetate using Et_3_N or NaOEt in ethanol under conventional heating for 8–10 h or MWI for 3 min to create 6-ethoxy-7*H*-[1,2,4]triazolo[3,4-*b*][1,3,4]thiadiazine **13**. When microwave irradiation (MWI) was used, the yield was 91–94%, but conventional heating only resulted in a yield of 79–83%. Condensation of **1** with bromoacetyl bromide or chloroacetic acid in MeOH containing Et_3_N or AcONa under conventional heating for 6 h or MWI for 2–3 min yielded 3-(3-fluorophenyl)-5,7-dihydro-1,2,4-triazolo[3,4-*b*][1,3,4]thiadiazin-6-one **14** in 85% and 95% or 78%, and 89% yields, respectively [26] (Scheme 5).

**Scheme 6:**
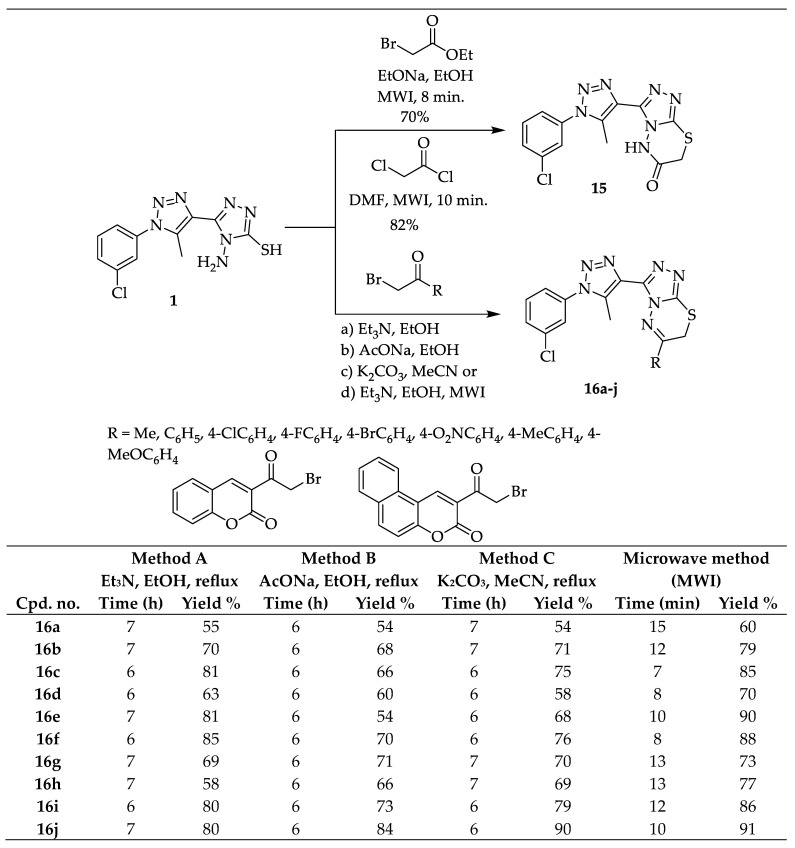
Synthesis of triazolo[3,4-*b*]thiadiazines derivatives **15** and **16**.

Compound **1** interacted with various α-halocarbonyl compounds in DMF with sodium ethoxide or microwave irradiation for 8 h and 10 min, respectively, to generate triazolo[3,4-*b*]thiadiazines **15** and **16a–j** (Scheme 6). The latter compounds have significant antimicrobial activity [27].

**Scheme 7:**
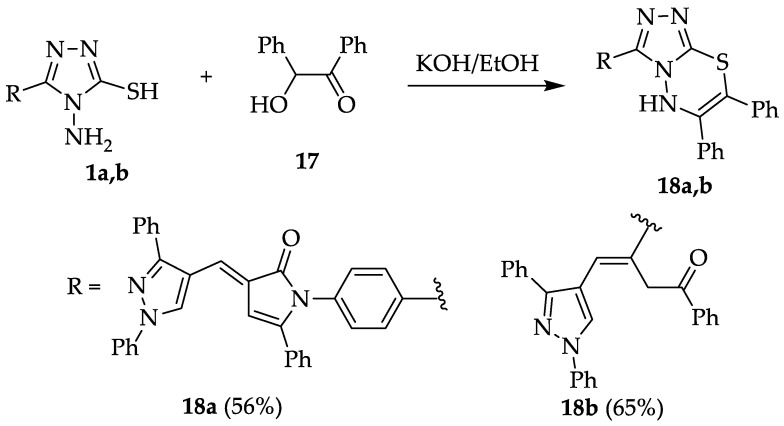
Synthesis of 6,7-diphenyl-5*H*-[1,2,4]triazolo[3,4-*b*][1,3,4]thiadiazine derivatives **18a**,**b**.

Compound **1** was subjected to benzoin treatment, resulting in the formation of 6,7-diphenyl-5*H*-[1,2,4]triazolo[3,4-*b*][1,3,4]thiadiazine derivatives **18a**,**b** [8,10] (Scheme 7). The produced compounds were assessed for their anticancer and antibacterial properties. Several of the investigated compounds exhibited encouraging actions [8]. Moreover, the compounds were assessed for their antiviral activity against highly pathogenic avian influenza (HPAI H_5_N_1_) [10].

**Scheme 8:**
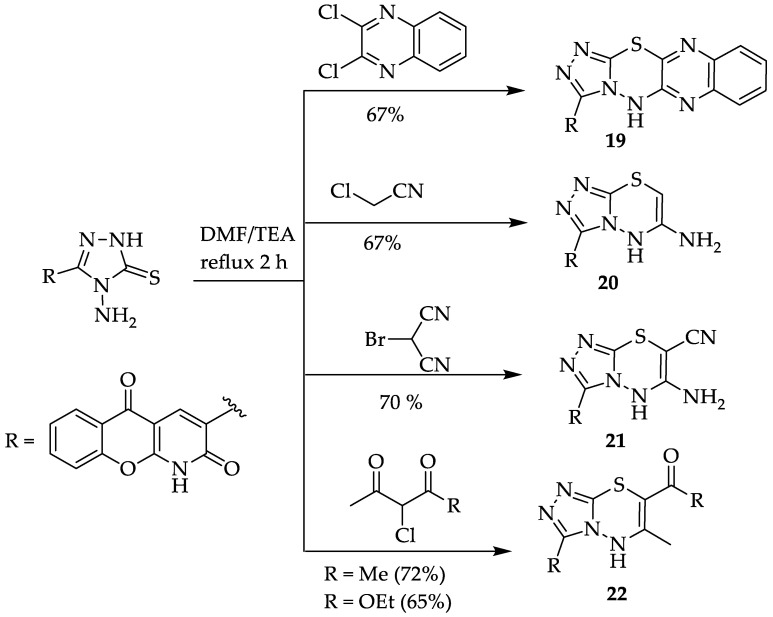
Synthesis of triazolo[3,4-*b*][1,3,4]thiadiazine derivatives **19**, **20**, **21**, and **22**.

Triazolo[3,4-*b*][1,3,4]thiadiazine derivatives **19**–**22** were produced by reacting compound **1** with 2,3-dichloroquinoxaline, chloroacetonitrile, bromomalononitrile, 3-chloropentane-2,5-dione, and ethyl 2-chloro-3-oxobutanoate in boiling DMF/TEA, respectively (Scheme 8). The acquired compounds were assessed for their antibacterial efficacy, displaying varying levels of inhibitory activity against the tested pathogens [28].

**Scheme 9:**
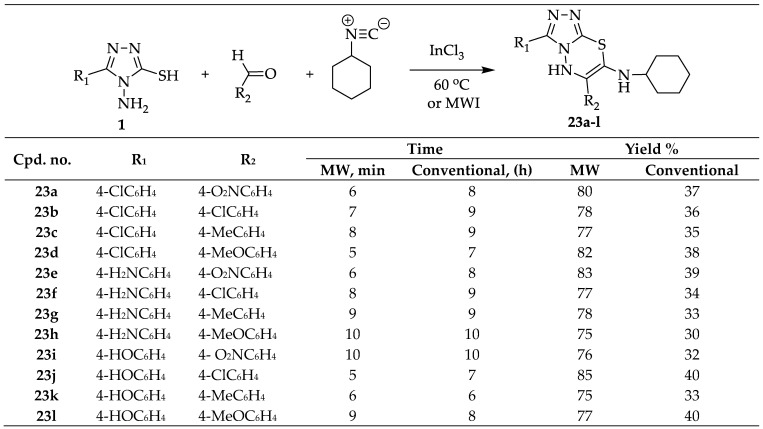
Synthesis of triazolothiadiazines **23a**–**l.**

In an alcoholic solution containing InCl_3_ as a recyclable catalyst, compound **1**, aromatic aldehyde, and isocyanocyclohexane were stirred at 60 °C or subjected to MWI to afford triazolothiadiazines **23a–l** [29] (Scheme 9).

**Scheme 10:**
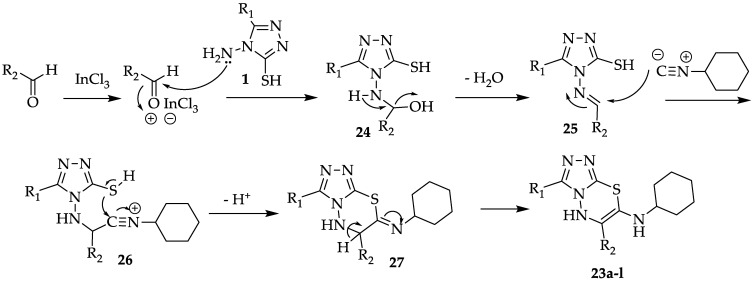
Mechanism of formation of triazolothiadiazines **23a**–**l.**

Synthesis of triazolothiadiazine **23** involved a chemoselective sequential double addition reaction. This included an InCl_3_-catalyzed dehydrative nucleophilic addition of an amino group to an aromatic aldehyde, followed by the addition of the resulting Schiff’s base **25** to cyclohexyl isocyanide. Adduct **26** undergoes intramolecular cyclization to produce the required molecules [29] (Scheme 10).

**Scheme 11:**
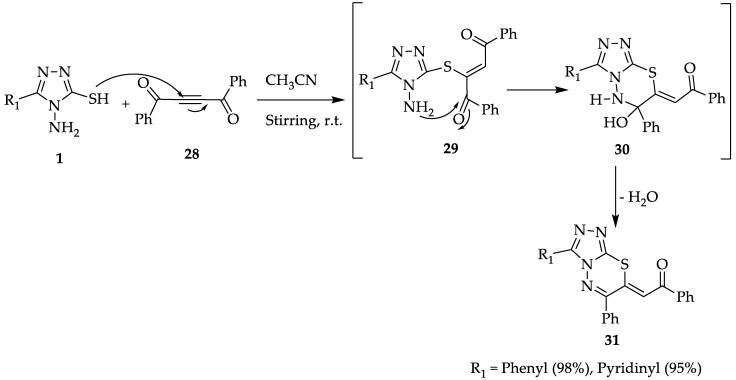
Synthesis of triazolo[3,4-*b*][1,3,4]thiadiazines **31.**

Triazolo[3,4-*b*][1,3,4]thiadiazines **31** were produced with high efficiency by a catalyst-free one-pot reaction between compound **1** and dibenzoylacetylene **28**. Single crystal X-ray diffraction was used to determine the structures of the generated molecules [30] (Scheme 11).

**Scheme 12:**
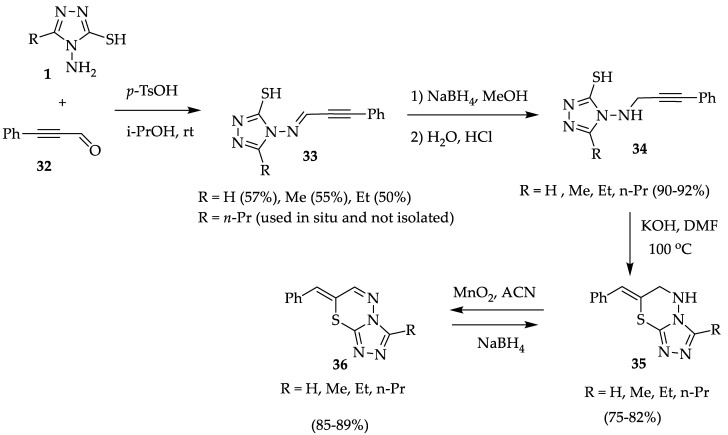
Synthesis of [1,2,4]triazolo[3,4-*b*][1,3,4]thiadiazines **36**.

3-Phenylpropiolaldehyde **32** was reacted with **1** in *i*-PrOH containing *p*-TsOH to give 4-((3-phenylprop-2-yn-1-ylidene)amino)-4*H*-1,2,4-triazole-3-thiol **33** then hydrogenation using sodium borohydride in methanol to afford **34**. Intramolecular cyclization of the latter compound in DMF with KOH as a catalyst at 100 °C afforded 3-substituted (Z)-7-benzylidene-6,7-dihydro-5*H*-[1,2,4]triazolo[3,4-*b*][1,3,4]thiadiazines **35**. Oxidation of **35** in acetonitrile using MnO_2_ gave [1,2,4]triazolo[3,4-*b*][1,3,4]thiadiazines **36**. However, hydrogenation **36** with sodium borohydride afforded **35** [11,31] (Scheme 12).

**Scheme 13:**
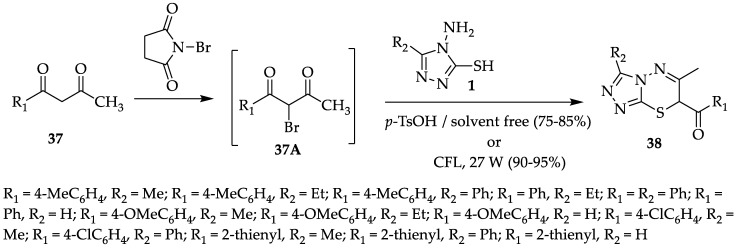
Synthesis of 7-aroyl-6-methyl-[1,2,4]triazolo[3,4-*b*][1,3,4]thiadiazines **38**.

A one-pot reaction is performed utilizing visible-light photoredox catalysis to react 1,3-diketones **37** with NBS and compound **1**. The reaction can be catalyzed by either *p*-TsOH or CFL (27 W) in a mixture of ethanol and water (4:1). The synthesis of 7-aroyl-6-methyl-[1,2,4]triazolo[3,4-*b*][1,3,4]thiadiazines **38** was achieved [32] (Scheme 13).

**Scheme 14:**
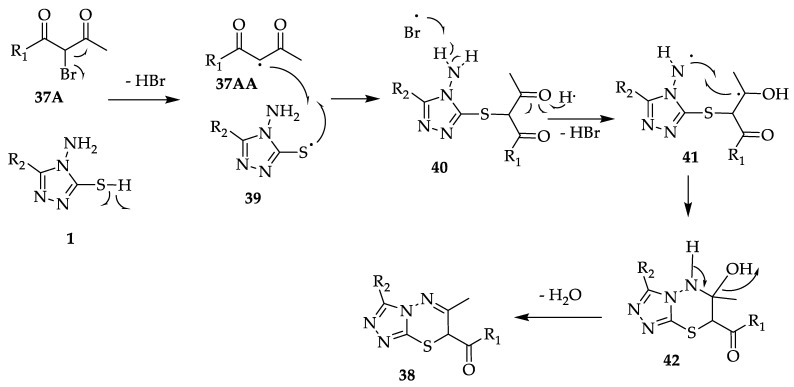
Mechanism of formation of triazolothiadiazines **38**.

The plausible mechanism for the regioselective production of **38** was detailed in Scheme 14. The C-Br bond of α-bromodiketone **37A** and the S-H bond of compound **1** underwent homolytic fission when exposed to visible light. This resulted in the formation of free radicals **37AA** and **39**. The radicals were combined to create an *S*-alkylated open chain intermediate **40**. After the N-H bond is broken homolytically with the help of a Br radical and the carbonyl carbon is cleaved with the assistance of a hydrogen free radical, there is a promotion of intramolecular free radical combination between the amine and the carbonyl carbon near the methyl group. Removing water led to the successful production of the desired product [32] (Scheme 14).

**Scheme 15:**
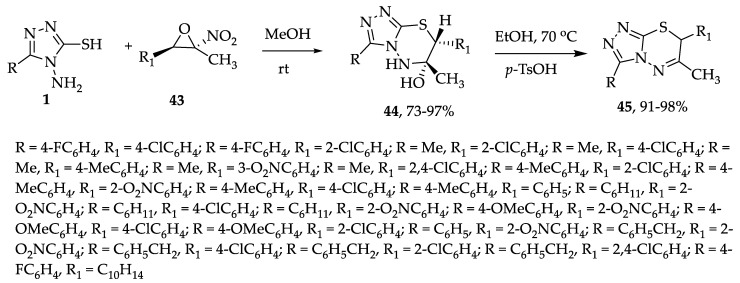
Synthesis of [1,2,4]triazolo[3,4-*b*][1,3,4]thiadiazines **45**.

A very effective synthesis of triazolothiadiazinols **44** was produced using a catalyst-free method. The synthesis involves the reaction of compound **1** with substituted nitroepoxide **43** in methanol at an ambient temperature, resulting in excellent regio- and diastereoselectivity. The dehydration of compound **44** using *p*-TsOH in EtOH at 70 °C resulted in the formation of the corresponding [1,2,4]triazolo[3,4-*b*][1,3,4]thiadiazines [33] (Scheme 15).

**Scheme 16:**
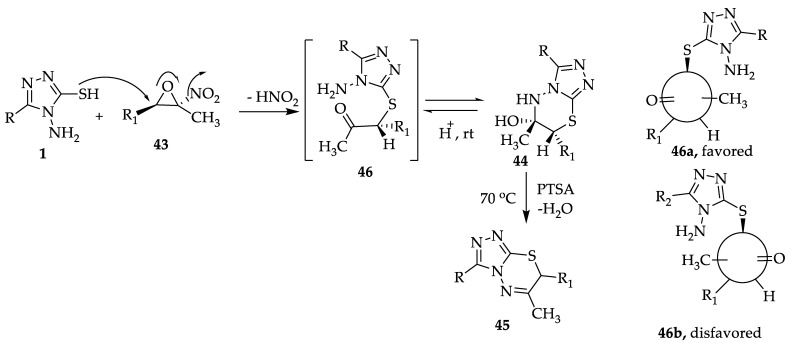
The mechanism of formation [1,2,4]triazolo[3,4-*b*][1,3,4]thiadiazines **45**.

Compound **45**′s synthesis process is depicted in Scheme 16. The thiol group opens the nitroepoxide ring, leading to the creation of intermediate **46** by eliminating nitric acid. The amino group then reacts via a nucleophilic attack on the carbonyl, leading to the creation of triazolothiadiazinol **44**. The stereochemistry of the compounds is explained using two Newman projections (**46a** and **46b**). The reaction proceeded through conformer **46a** due to the unfavorable contact between the methyl and aryl groups in **46b**, resulting in the products with the observed stereochemistry [33].

**Scheme 17:**
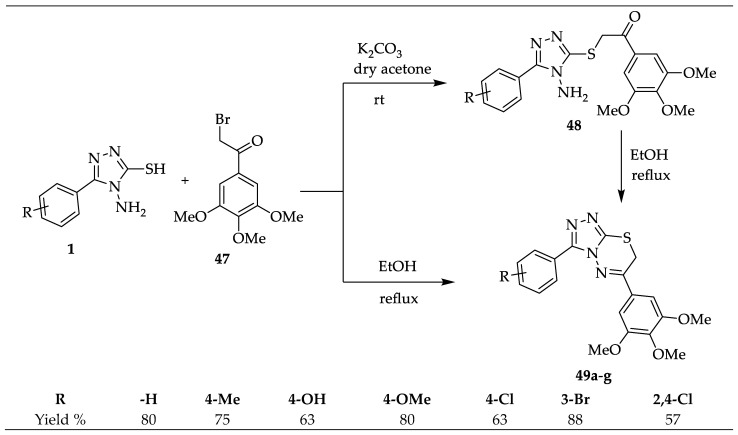
Synthesis of triazolothiadiazines **49a**–**g.**

The molecule **1** was alkylated with 2-bromo-1-(3,4,5-trimethoxyphenyl)ethan-1-one **47** in dry acetone with K_2_CO_3_ to produce *S*-phenacyl derivatives **48**. When the subsequent reaction was carried out in refluxing ethanol, it produced the cyclized analogs **49**. In addition, the annulation of **48** was achieved by subjecting it to reflux in absolute ethanol, resulting in the formation of **49** (Scheme 17). The produced compounds exhibited remarkable selectivity towards cancer cells compared to normal cells, since they did not demonstrate any notable cytotoxicity against L929 cells. Furthermore, compound **49a** has the ability to hinder the process of tubulin polymerization at micro-molar concentrations [12].

**Scheme 18:**
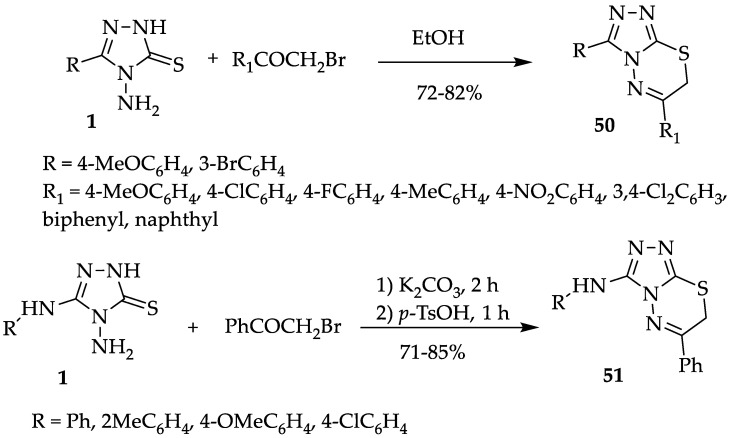
Synthesis of triazolothiadiazines **50** and **51**.

Similarly, triazolothiadiazines **50** were achieved by condensing **1** with phenacyl bromides in refluxing ethanol, as reported [34]. Triazolothiadiazines **51** were produced by reacting **1** with *α*-bromoacetophenone in the presence of K_2_CO_3_ and *p*-TsOH [35] (Scheme 18). The synthesized compounds were assessed as very effective and specific inhibitors of electric eel acetylcholinesterase (EeAChE) and horse serum butyrylcholinesterase (hBChE) using Ellman’s method, with neostigmine and donepezil serving as standard inhibitors.

**Scheme 19:**
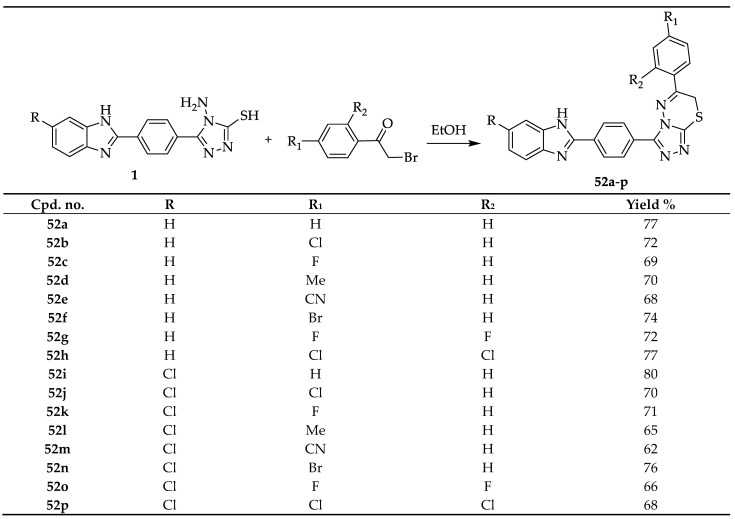
Synthesis of benzimidazole-triazolothiadiazine derivatives **52a**–**p.**

In the same manner, substituted phenacyl bromide was reacted with compound **1** to give benzimidazole-triazolothiadiazine derivatives **52** (Scheme 19). The synthesized compounds exhibited anticancer efficacy against the MCF-7 human breast cancer cell lines. Compounds **52c**, **52e**, **52k**, and **52m**, which showed the highest activity on the MCF-7 cell line, were selected for additional in vitro experiments to investigate the potential mechanisms responsible for their activity. These assays focused on determining their ability to inhibit the aromatase enzyme [13].

**Scheme 20:**
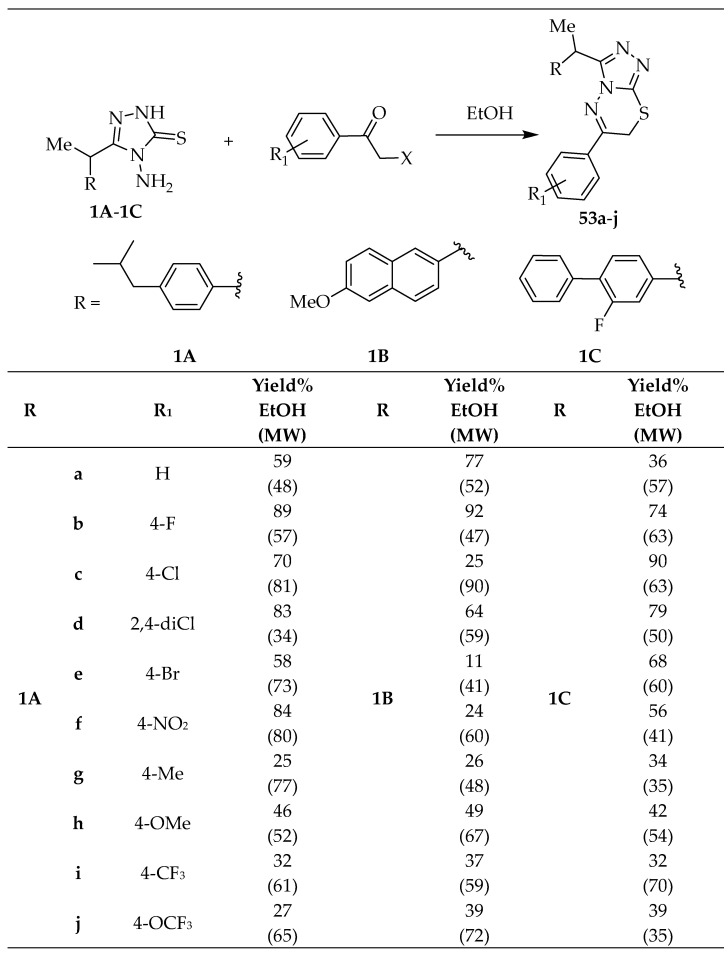
Synthesis of 1,2,4-triazolo[3,4-*b*]-1,3,4-thiadiazines **53a**–**j**.

In addition, 4-amino-3-[1-[4-(substituted phenyl]ethyl]-1,2,4-triazole-5-thiones **1I–3I** were condensed with phenacyl halides in reflux ethanol or under microwave irradiation (350 Watt) to give 1,2,4-triazolo[3,4-*b*]-1,3,4-thiadiazines **53a**–**j** (Scheme 20). The produced compounds were screened for their bioactivity against epithelial cancer cells and this demonstrated that those derivatives are promising drug candidates for epithelial cancers, especially liver cancer [36].

**Scheme 21:**
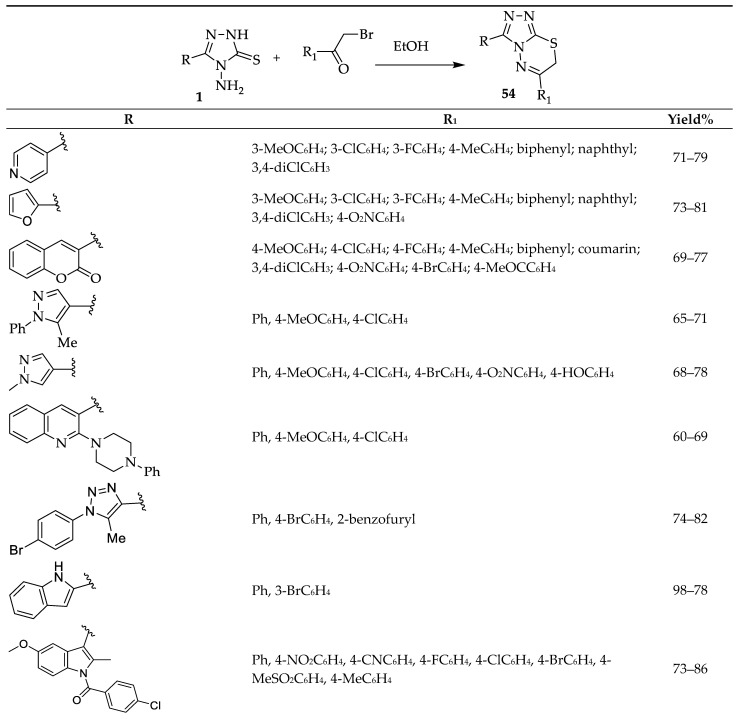

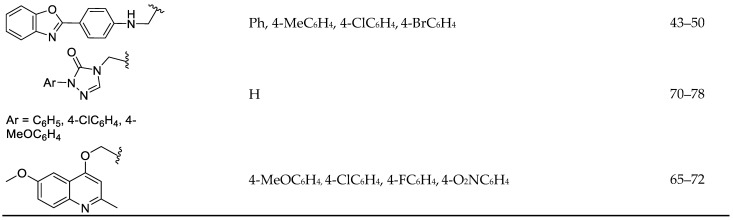
Synthesis of 1,2,4-triazolo[3,4-*b*]-1,3,4-thiadiazines **54** [37,38,39,40,41,42,43,44,45,46,47,48,49].

The heterocyclic derivatives of **1** were subjected to treatment with substituted phenacyl bromides in pure ethanol. This resulted in the formation of 1,2,4-triazolo[3,4-*b*][1,3,4]thiadiazine derivatives **54** [37,38,39,40,41,42,43,44,45,46,47,48,49] (Scheme 21). The produced compounds underwent screening to assess their inhibitory effects on acetyl- and butyryl-cholinesterases, as well as alkaline phosphatase. The majority of compounds exhibited superior efficacy against acetylcholinesterase compared to the reference medicines [37]. In addition, their antiviral efficacy against the reproduction of both HIV-1 and HIV-2 in MT-4 cells was assessed using an MTT assay [38]. The prepared compounds were assessed for their effects on alkaline phosphatase (ALP), tested against Leishmania major, and their anticancer activity was determined using kidney fibroblast (BHK-21) and lung carcinoma (H-157) cancer cell lines [39]. Additionally, the inhibitory potential against both tissue-nonspecific alkaline phosphatase (h-TNAP) and intestinal alkaline phosphatase (h-IAP) were investigated, with the aim of establishing a connection between their alkaline phosphatase inhibitory properties and potential anti-proliferative and pro-apoptotic effects. The examined compounds had significant inhibitory effects on h-TNAP and h-IAP enzymes, surpassing the inhibitory activity of conventional medicines [40]. Additionally, their antibacterial activity against four human pathogenic bacteria were evaluated: 6-(4-Methoxyphenyl)-3-(5-methyl-1-phenyl-1*H*-pyrazol-4-yl)-7*H*-[1,2,4]triazolo[3,4-*b*][1,3,4]thiadiazine demonstrates significant efficacy against all bacteria [41]. The synthesized compounds were assessed for their in vitro antiproliferative properties against MCF-7 and MDA-MB-231 breast cancer cell lines using the MTT test [47]. Furthermore, the synthesized compounds were assessed for their in vitro antibacterial activity. The majority of compounds showed a satisfactory level of antibacterial activity and are notably more potent than ampicillin [49].

**Scheme 22:**
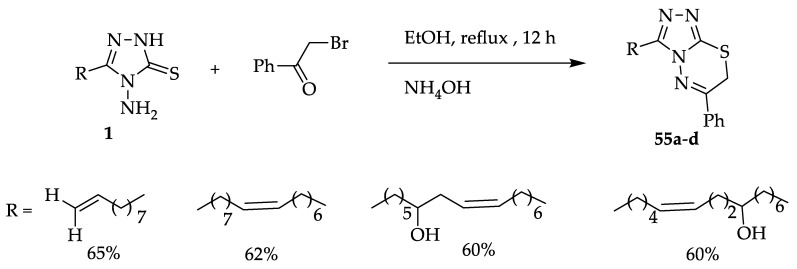
Synthesis of long chain triazolothiadiazines **55a**–**d.**

Long chain alkenyl-6-phenyl-7*H*-1,2,4-triazolo[3,4-*b*]-1,3,4-thiadiazines **55a**–**d**, were synthesized from the reaction of compound **1** and phenacyl bromide via a ring closure reaction (Scheme 22). The prepared compounds are promising anticancer agents [50].

**Scheme 23:**
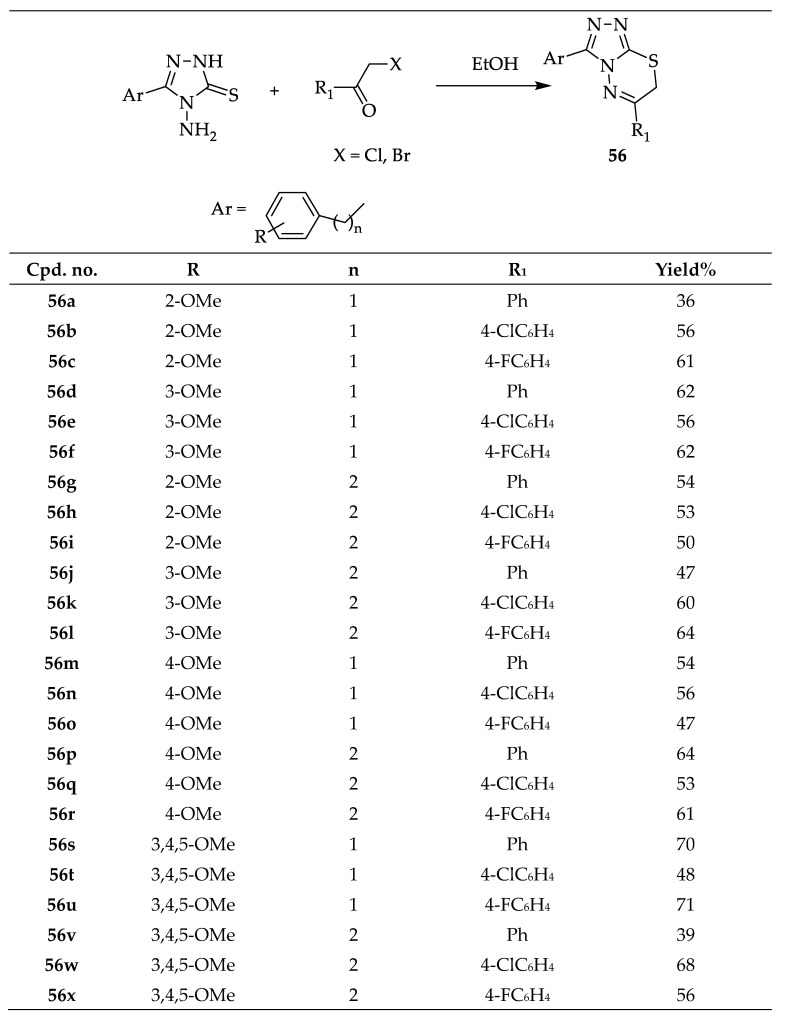
Synthesis of triazolothiadiazine derivatives **56.**

In the same fashion, triazolothiadiazine derivatives **56** were synthesized through the reaction of **1** with phenacyl halides in EtOH at reflux conditions [14,15,51] (Scheme 23). The synthesized compounds were tested for their ability to relieve pain and reduce inflammation. Also, all compounds were tested for their ability to harm the digestive system and their antioxidant activity. Most of the substances worked well in both the carrageenan-induced oedema and acetic acid-induced writhing tests, with only minor effects on the digestive system. Most successful results were seen with molecules that added a chlorine or fluorine atom to the sixth position of the fused phenyl ring [15]. The effects of triazolothiadiazines on hepatocellular carcinoma (HCC) cells were described. Compound **56t** had the highest efficacy in halting the cell cycle specifically in the G2/M phase and inducing cell death in HCC cells. Oxidative stress induced activation of the JNK protein, resulting in a reconfiguration of the roles of ASK1, MKK7, and c-Jun proteins. In addition, nude mice treated with **56t** had reduced growth tissue and experienced an extended lifespan without succumbing to illness. Additionally, **56t** hindered the movement of HCC cells and the accumulation of liver cancer stem cells (LCSCs), both individually and when used in conjunction with sorafenib. Compound **56t** has the potential to impact the proliferation, stemness, and migration of HCC cells, making it a possible treatment option for this increasingly prevalent disease, which is seeing an annual growth rate of approximately 3% [51].

**Scheme 24:**
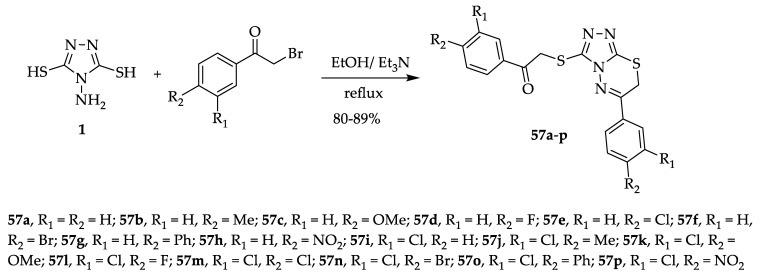
Synthesis of 1-Aryl-2-((6-aryl-7*H*-[1,2,4]triazolo[3,4-*b*][1,3,4]thiadiazin-3-yl)thio)ethan-1-ones **57a**–**p**.

1-Aryl-2-((6-aryl-7*H*-[1,2,4]triazolo[3,4-*b*][1,3,4]thiadiazin-3-yl)thio)ethan-1-one derivatives **57a–p** were produced from a reaction between **1** and variously substituted phenacyl bromides in ethanol containing Et_3_N (Scheme 24). The compounds underwent screening to evaluate their in vitro anticancer efficacy against a panel of 60 cancer cell lines. Compound **57d**, **57f**, and **57l** showed substantial efficacy in inhibiting the growth of the kidney cancer OU-31 cell line, with growth percentages of 47.42%, 46.76%, and 48.14% respectively. Compound **57l** demonstrated noteworthy efficacy against the leukemia MOLT-4 cell line, resulting in a reduction of 49.82% [52].

**Scheme 25:**
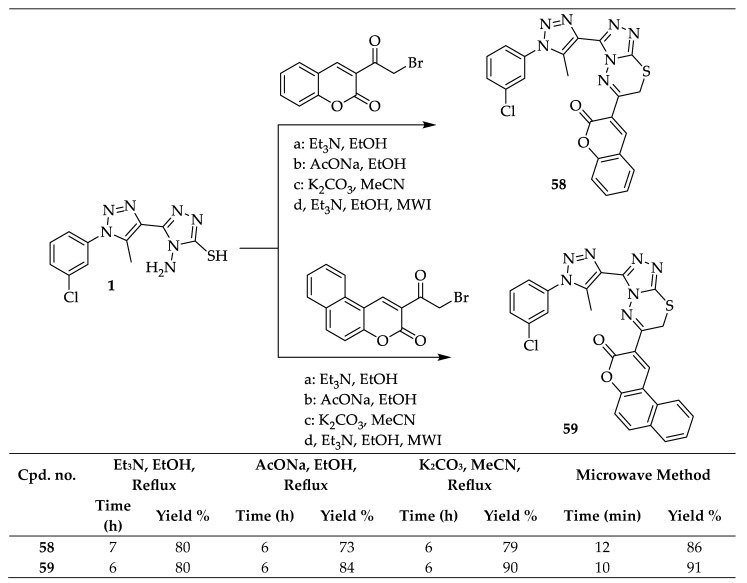
Synthesis of triazolo[3,4-*b*]thiadiazines **58** and **59**.

Triazolo[3,4-*b*]thiadiazines **58** and **59** were produced from the reaction of **1** with 3-(2-bromoacetyl)-2*H*-chromen-2-one and 2-(2-bromoacetyl)-3*H*-benzo[*f*]chromen-3-one under either the conventional or microwave conditions. The most favorable outcome was achieved by utilizing microwave conditions [27] (Scheme 25).

**Scheme 26:**
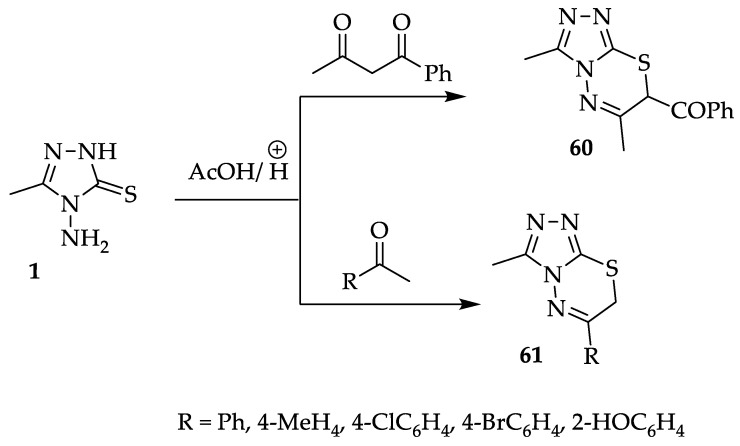
Synthesis of triazolothiadiazines **60** and **61.**

When **1** was treated with 1-phenylbutane-1,3-dione and aromatic ketones in glacial acetic acid with drops of conc. sulfuric acid as a catalyst, it produced 7-benzoyl-3,6-dimethyl-s-triazolo[3,4-*b*]1,3,4-thiadiazine **60** and 3-methyl-7*H*-s-triazolo[3,4-*b*]-1,3,4-thiadiazines **61**, respectively (Scheme 26). The synthesized compounds show significant potential for eliminating heavy metal ions and inorganic anions from water-based systems. The metal ion removal efficiency achieved a high level of 76.29%, whereas the removal efficiency for inorganic anions reached a perfect 100%. These findings indicate that the synthesized compounds possess significant potential as adsorbents for the purpose of water filtration [53].

**Scheme 27:**
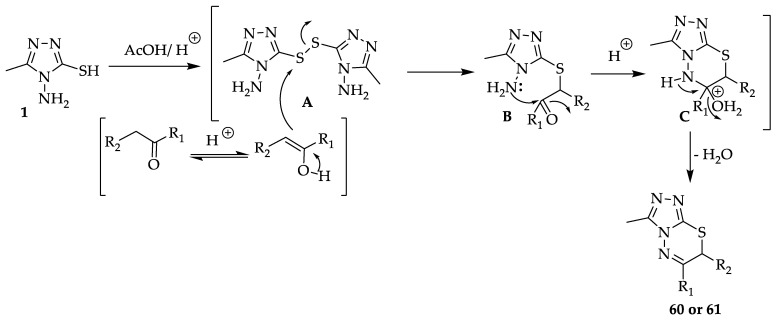
Mechanism of formation of triazolothiadiazines **60** and **61**.

**Scheme 28:**
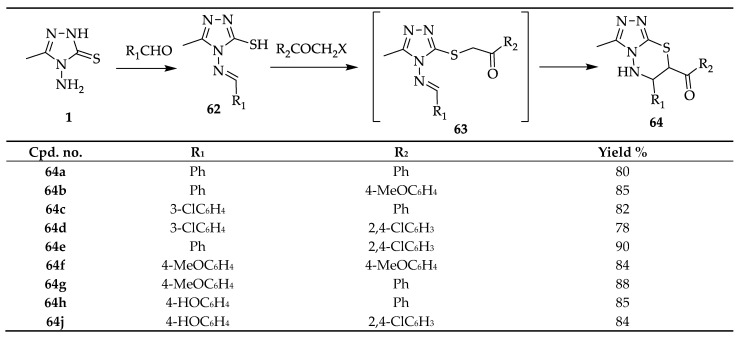
Synthesis of triazolo[3,4-b][1,3,4]thiadiazinyl methanones **64**.

The proposed mechanisms for the formation of compounds **60** and **61** are summarized in Scheme 27. The s-triazole is initially oxidized to form the disulfide intermediate **A**, which is followed by the nucleophilic attack of the enolate form of the ketone to give the S-alkylation intermediate **B**. This formed intermediate then undergoes intramolecular cyclization to yield the desired products. 

(*E*)-4-(Arylideneamino)-5-methyl-4*H*-1,2,4-triazole-3-thiol **62** was treated with appropriate substituted *ω*-bromoacetophenone in EtOH containing Et_3_N at room temperature to give (3-methyl-6-aryl-6,7-dihydro-5*H*-[1,2,4]triazolo[3,4-*b*][1,3,4]thiadiazin-7-yl)(aryl)methanones **64** via intermediate **63** (Scheme 28). The synthesized compounds were assessed for their antiproliferative activity against HepG2 cell lines in vitro. Additionally, their effects on plant growth regulation were evaluated on wheat and radish. The results revealed that all of the synthesized compounds showed a remarkably low antiproliferative activity against HepG2 cell lines in vitro, contrary to expectations. However, they demonstrated significant plant growth-regulating effects on both wheat and radish [54].

**Scheme 29:**
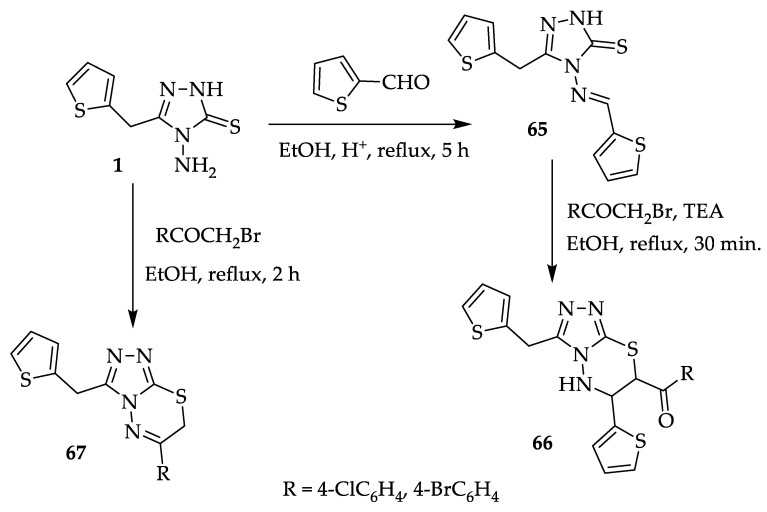
Synthesis of fused heterocycle triazolothiadiazines **66** and **67.**

Aminotriazolethione **1** was reacted with thiophene-2-carbaldehyde to afford the corresponding Schiff’s base **65**. The latter compound was then treated with phenacyl bromides leading to fused heterocycle triazolothiadiazines **66**. Moreover, compounds **67** were obtained directly by the reaction of **1** with phenacyl bromides [55] (Scheme 29).

### 2.2. From 4-Amino-5-hydrazinyl-4H-1,2,4-triazole-3-thiol (Purpald)

**Scheme 30:**
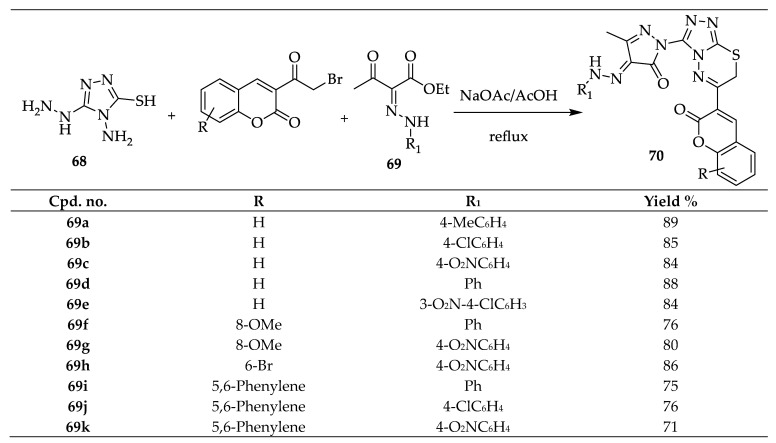
Synthesis of triazolothiadiazinyl pyrazol-3-one derivatives **70**.

A one-pot, multicomponent process involving 3-(2-bromoacetyl)coumarin derivatives, 4-amino-5-hydrazino-4*H*-[1,2,4]triazole-3-thiol **68**, and ethyl-2-(2- arylhydrazono)-3-oxobutanoate **69** in acetic acid with sodium acetate to yield 2-(7*H*-[1,2,4]triazolo[3,4-*b*][1,3,4]thiadiazin-3-yl)-5-methyl-2,4-dihydro-3*H*-pyrazol-3-one derivatives **70** was reported [56] (Scheme 30).

**Scheme 31:**
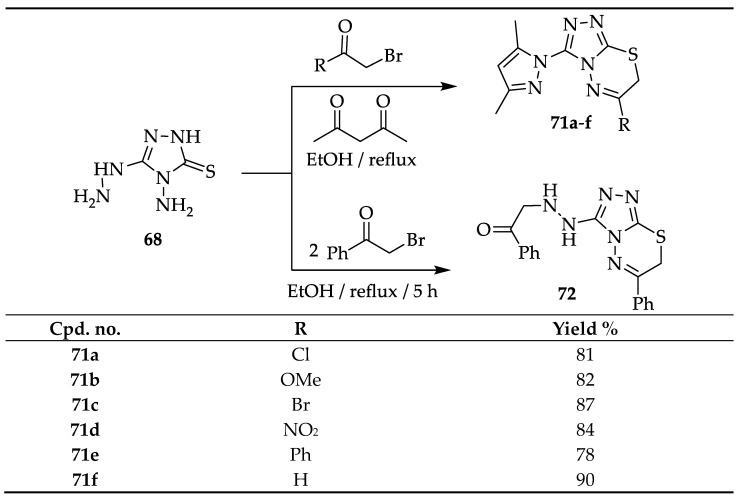
Synthesis of triazolo[3,4-*b*]thiadiazines **71** and **72**.

On the other hand, a one-pot reaction of **68**, phenacyl bromides, and acetyacetone in EtOH at reflux gave 3-(3,5-dimethyl-1*H*-pyrazol-1-yl)-7*H*-[1,2,4]triazolo[3,4-*b*][1,3,4]thiadiazines **71**, while reacting compound **68** with two moles of phenacyl bromide yielded *N*′-(6-phenyl-7*H*-[1,2,4]triazolo[3,4-*b*][1,3,4]thiadiazin-3-yl)benzohydrazide **72** [56,57] (Scheme 31).

**Scheme 32:**
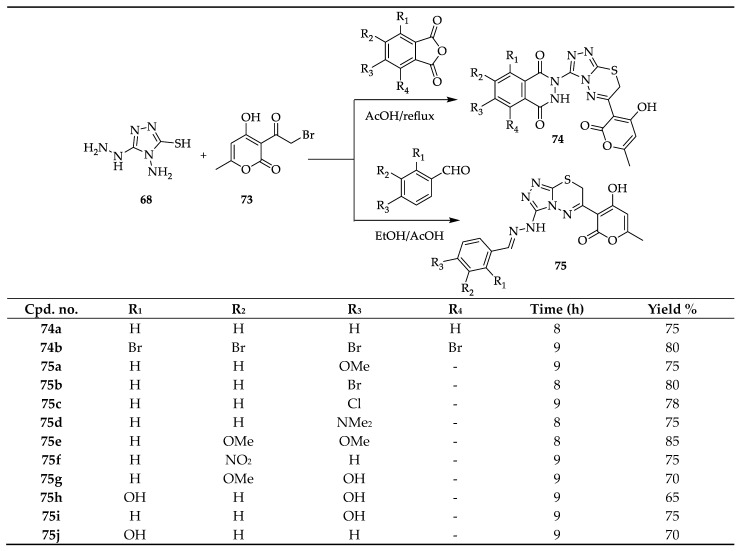
Synthesis of triazolothiadiazinyl dihydrophthalazine-1,4-diones **74** and triazolothiadiazine hydrazone **75.**

A one-pot, three-components reaction of Purpald **68**, 3-(2-bromoacetyl)-4-hydroxy-6-methyl-2*H*-pyran-2-one **73** and phthalic anhydride in AcOH at reflux afforded 2-(6-(4-hydroxy-6-methyl-2-oxo-2*H*-pyran-3-yl)-7*H*-[1,2,4]triazolo[3,4-*b*][1,3,4]thiadiazin-3-yl)-2,3-dihydrophthalazine-1,4-diones **74**, while triazolothiadiazine hydrazone derivatives **75** were produced from a one pot reaction of **68**, **73**, and substituted benzaldehyde [58,59] (Scheme 32).

**Scheme 33:**
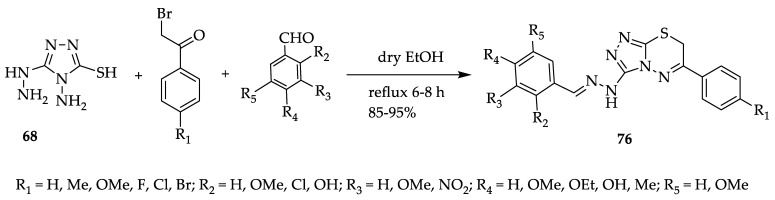
Synthesis of triazolothiadiazine hydrazone derivatives **76**.

On the other hand, a series of triazolothiadiazine hydrazone derivatives **76** was synthesized via a one-pot, three-components reaction of Purpald **68**, phenacyl bromides, and various aromatic aldehydes in EtOH at a reflex temperature [60,61] (Scheme 33).

**Scheme 34:**
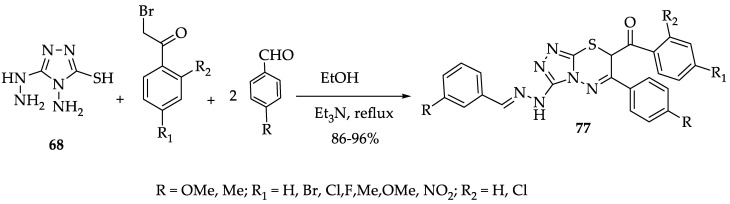
Synthesis of triazolothiadiazine hydrazone derivatives **77**.

Similarly, triazolothiadiazine hydrazone derivatives **77** were produced via a one-pot four component approach involving the condensation of **68**, aromatic aldehydes and various phenacyl bromides in absolute EtOH containing Et_3_N [62] (Scheme 34).

**Scheme 35:**
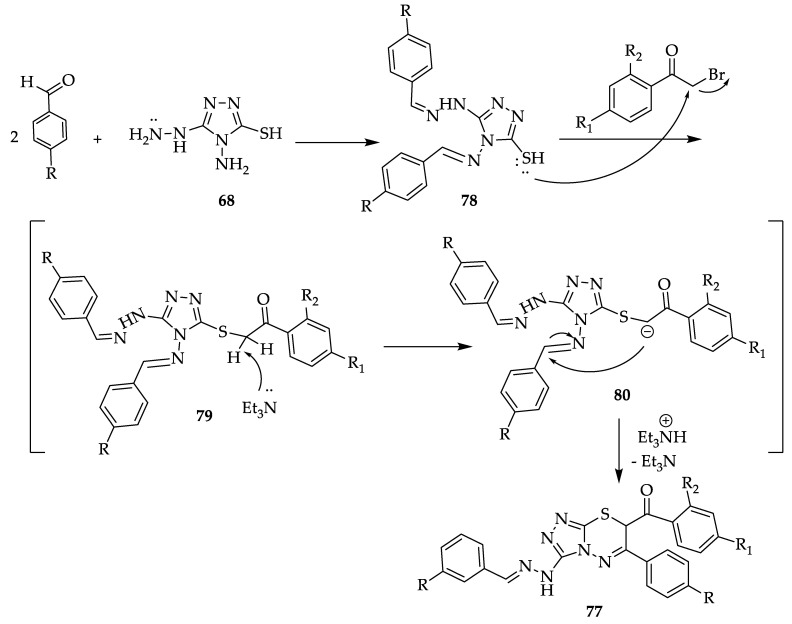
Mechanism of formation of hydrazone derivatives **77**.

**Scheme 36:**
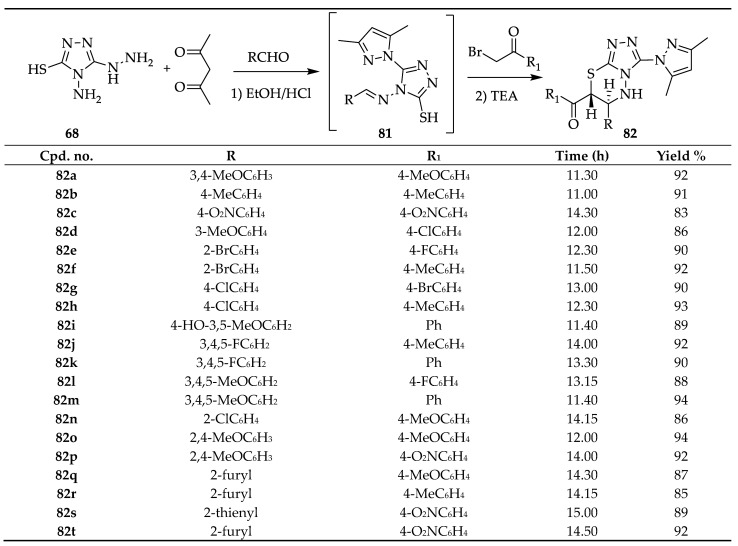
Synthesis of 3-pyrazolyl triazolothiadiazines **82**.

The mechanism of producing compound **77** is described in the Scheme. Firstly, dianils **78** were formed; then a nucleophilic substitution reaction between the latter dianils and phanacyl bromide was followed by an annulation reaction via intermediates **79** and **80** [62] (Scheme 35).

(±)-3-(1*H*-Pyrazol-1-yl)-6,7-dihydro-5*H*-[1,2,4]triazolo[3,4-*b*][1,3,4]thiadiazines **82** were developed using a two-step, one pot four-components reaction of **68**, acetylacetone, aldehyde in EtOH with drops of conc. HCl at reflux to give 4-(arylideneamino)-5-(3,5-dimethyl-1*H*-pyrazol-1-yl)-4*H*-1,2,4-triazole-3-thiol **81**, not isolated, then substituted phenacyl bromide and Et_3_N were added. Then the mixture was refluxed to produce the target compounds (Scheme 36). The prepared compounds were tried to see how well they worked against viruses and tumors. It was shown that small changes in the structure of the phenyl group could change the biological qualities to make them more effective against viruses or tumors. These compounds have the ability to fight tumors due to stopping tubulin from polymerizing [63].

### 2.3. From Thiocarbohydrazide

**Scheme 37:**
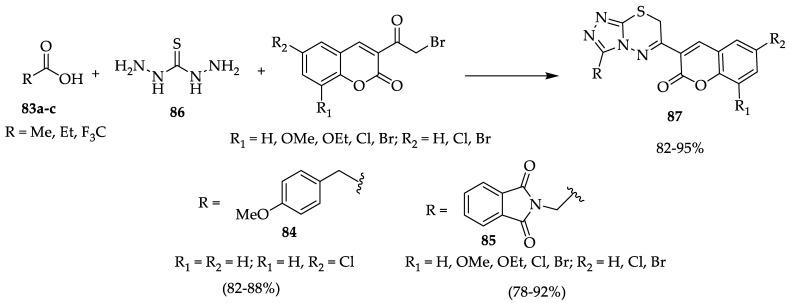
Synthesis of triazolo thiadiazinyl coumarins **87.**

The triazolo thiadiazinyl coumarins **87** were produced by a solvent-free one-pot multi-component reaction involving aliphatic **83a–c**, 2-(4-methoxyphenyl)acetic acid **84**, or 2-(1,3-dioxoisoindolin-2-yl)acetic acid **85**, thiocarbohydrazide **86**, and substituted 3-(2-bromoacetyl)coumarins (Scheme 37). The triazolo thiadiazinyl coumarins had remarkable antibacterial efficacy [64].

**Scheme 38:**
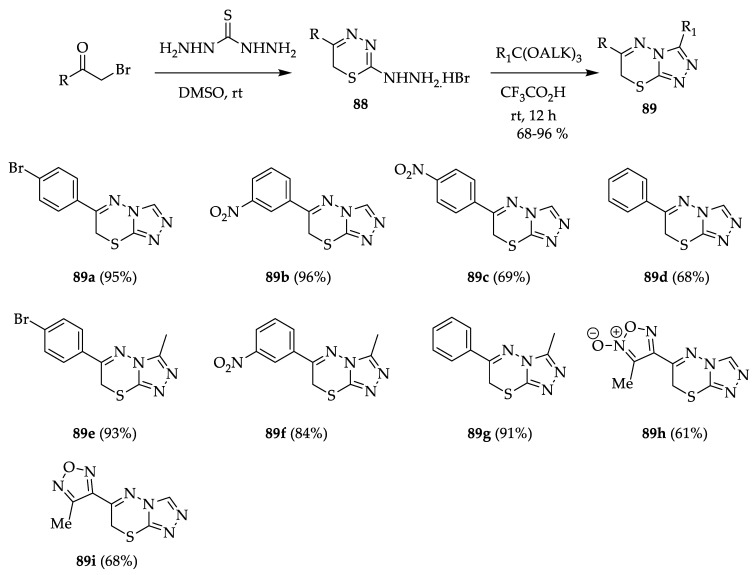
Synthesis of 1,2,4-triazolo[3,4-*b*][1,3,4]thiadiazines **89a**–**i**.

The reaction between *α*-bromo ketones and thiocarbohydrazide in DMSO at room temperature resulted in the formation of 2-hydrazinyl-6*H*-1,3,4-thiadiazine hydrobromides **88**. The latter compound underwent annulation with trimethyl orthoformate or triethyl orthoacetate in trifluoroacetic acid resulting in the formation of 1,2,4-triazolo[3,4-*b*][1,3,4]thiadiazines **89a–i** [65] (Scheme 38).

### 2.4. From 3-Methyl-5-(arylthio)-4H-1,2,4-triazol-4-amine

**Scheme 39:**
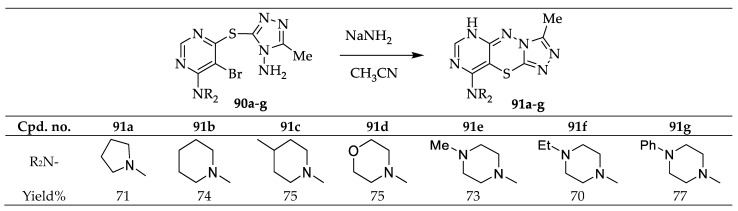
Synthesis of pyrimido[4,5-*e*][1,2,4]triazolo[3,4-*b*][1,3,4]thiadiazines **91a**–**g**.

An intramolecular S_N_Ar type, Smiles rearrangement, of 3-((5-bromo-6-(substituted)pyrimidin-4-yl)thio)-5-methyl-4*H*-1,2,4-triazol-4-amine in boiling acetonitrile in the presence of NaNH_2_ afforded 3-methyl-6-(substituted)-6*H*-pyrimido[4,5-*e*][1,2,4]triazolo[3,4-*b*][1,3,4]thiadiazines **91a–g** [66] (Scheme 39).

**Scheme 40:**
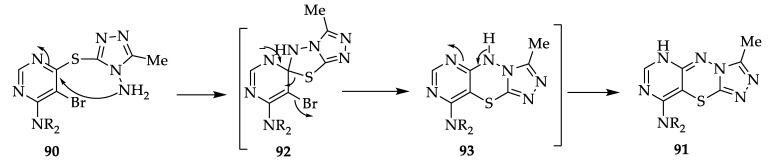
Mechanism of formation of triazolothiadiazines **91a–g**.

Scheme 40 illustrates a possible pathway for the creation of compound **91** via a Smiles-type rearrangement. The reaction begins with the creation of a spiro-type intermediate, which then rearranges and loses HBr to produce the desired product [66].

### 2.5. From 5-Substituted-1,2,4-triazole-3-thiol

**Scheme 41:**
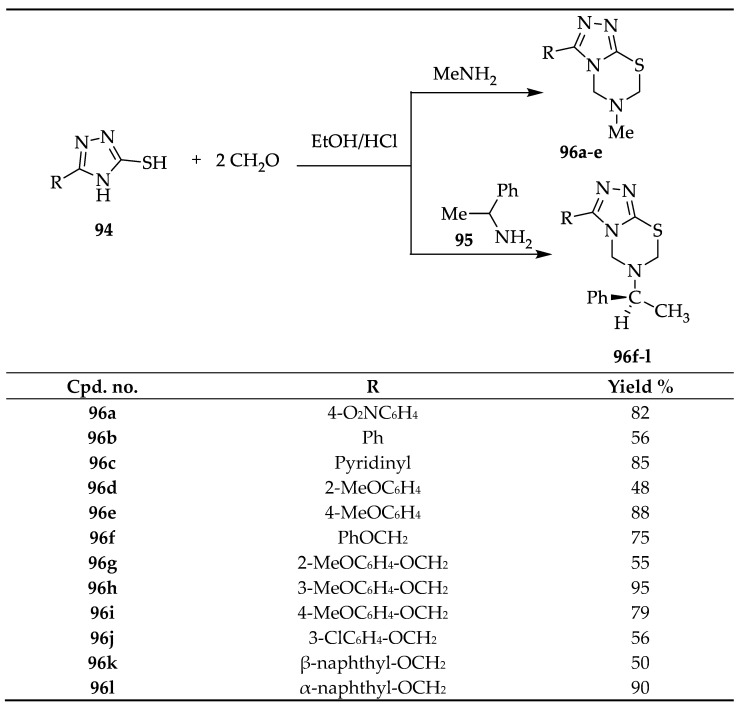
Synthesis of triazolothiadiazines **96a–l**.

A Mannich reaction of 1,2,4-triazole-3-thiol **94** with either methylamine [67] or S-(-)-α-phenylethylamine **95** [68] and formaldehyde, in EtOH using an acid catalyst, resulted in the formation of 1,2,4-triazolo[3,4-*b*][1,3,5]thiadiazines **96a**–**l** (Scheme 41).

## 3. Synthesis of Triazolo[5,1-*b*]thiadiazines

**Scheme 42:**
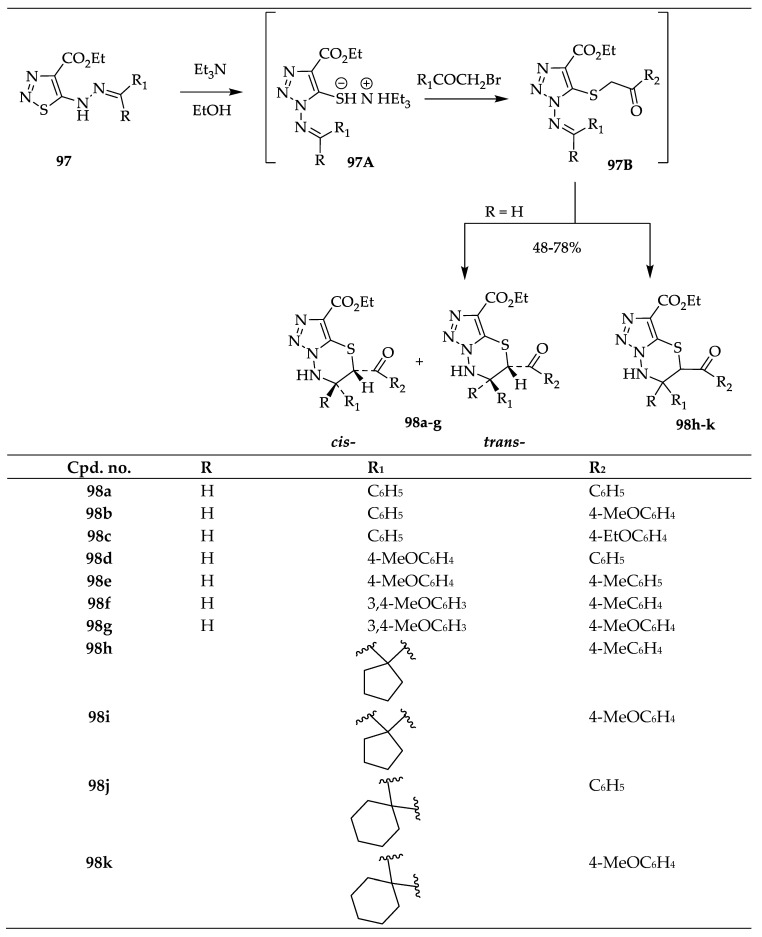
Synthesis of triazolo[5,1-*b*]thiadiazine-3-carboxylates **98a**–**k**.

A Dimroth rearrangement of 1,2,3-thiadiazolylhydrazones **97** give in situ 1,2,3-triazole-4-thiolates **97A**, was reacted with *α*-bromoacetophenones in ethanol with triethylamine; it produced *cis* and *trans*-isomers of ethyl-5-aroyl-6-aryl-6,7-dihydro-5*H*-[1,2,3]triazolo[5,1-*b*][1,3,4]thiadiazine-3-carboxylats **98a–g** in a 1:4 ratio [69]. Similarly, spirocyclic 1,2,3-triazolo[5,1-*b*]1,3,4-thiadiazines **98h–k** was synthesized from the corresponding 3-cyclopentylidene(cyclohexylidene)amino-triazole-4-thiolates [70] (Scheme 42). The crystal structures of **98h–k** were determined using single crystal X-ray diffraction [71].

**Scheme 43:**
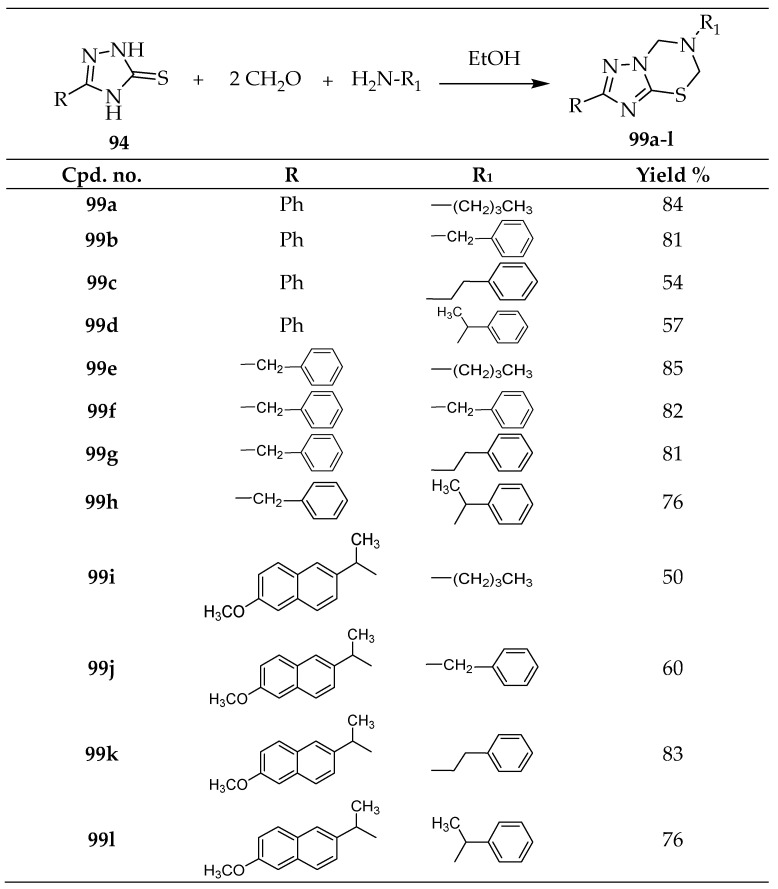
Synthesis of [1,2,4]triazolo[5,1-*b*][1,3,5]thiadiazines **99a**–**l**.

On the other hand, the prior mention [67,68]. The Mannich reaction involves the interaction of 1,2,4-triazole-5-thiones **94** with formaldehyde and different primary amines in ethanol. The investigation showed that the reaction proceeds by cyclization, using two moles of formaldehyde to produce [1,2,4]triazolo[5,1-*b*][1,3,5]thiadiazines **99a**–**l** [72] (Scheme 43).

## 4. Synthesis of Triazolo[4,3-*c*]thiadiazines

**Scheme 44:**
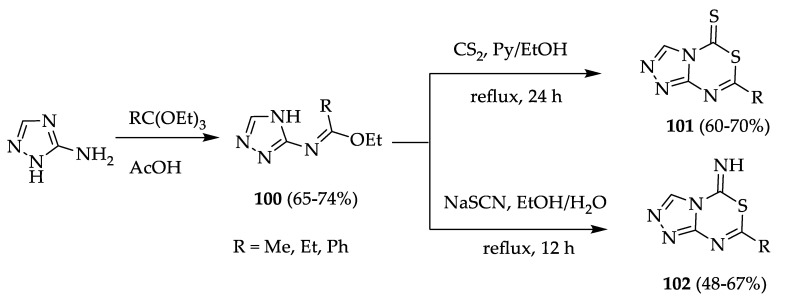
Synthesis of triazolo[4,3-*c*]thiadiazine-5-thiones **101** and triazolo[4,3-*c*]thiadiazin-5-imines **102**.

Condensation of 4*H*-1,2,4-triazol-3-amine with the ortho esters in AcOH affords carboximidates **100**. When compound **100** was treated with carbon disulfide in ethanol with pyridine at reflux temperature, it produced triazolo[4,3-*c*][1,3,5]thiadiazine-5-thiones **101**. Compound **100** was treated with sodium thiocyanate in a mixture of ethanol and water, heated under reflux for 12 h, resulting in the formation of triazolo[4,3-*c*][1,3,5]thiadiazin-5-imines **102** [73] (Scheme 44).

## 5. Synthesis of Triazolo[1,5-*c*]thiadiazines

**Scheme 45:**
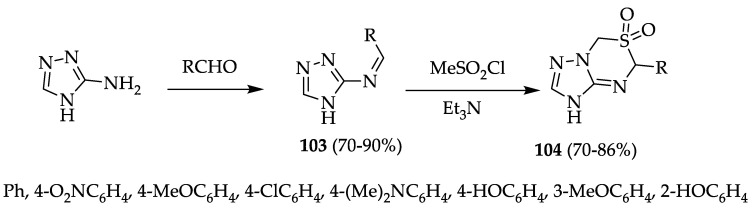
Synthesis of 3,5-dihydro-7*H*-[1,2,4]triazolo[1,5-*c*][1,3,5]thiadiazine 6,6-dioxides **104**.

3-Benzylidineamino-1,2,4-triazole **103** was synthesized by heating a mixture of 3-amino-l,2,4-triazole and suitable aldehydes in the presence of piperidine. A [4+2] cycloaddition reaction of **103** with methanesulfonyl chloride was performed in dioxane to afford [1,2,4]triazolo[3,2-c][1,3,5]-thiadiazine-3,3-dioxide derivatives **104** in a high yield (Scheme 45). The synthesized compounds underwent screening to evaluate their antifungal properties [74].

## 6. Conclusions

This survey aimed to provide a synopsis of the synthesis routes and pharmacological uses of triazolothiadiazine derivatives. Various methods were employed to synthesize triazolothiadiazines, such as (i) reacting specific compounds like 4-amino-5-mercapto-4*H*-1,2,4-triazole, 4-amino-5-hydrazinyl-4*H*-1,2,4-triazole-3-thiol (Purpald), or 5-(substituted thio)-4*H*-1,2,4-triazol-4-amine with different α-halocarbonyl compounds: hydrazonoyl halides, α-haloketone, α-haloester, α-haloacid, α-halonitrile, or with dibenzoylacetylene, benzoin, 3-phenylpropiolaldehyde, substituted nitroepoxide; (ii) conducting a one-pot reaction involving 4-amino-5-mercapto-4*H*-1,2,4-triazole, aromatic aldehyde, and isocyanocyclohexane using InCl_3_; (iii) engaging in a one-pot multi-component reaction of carboxylic acid or ortho ester, thiocarbohydrazide, and *α*-haloketone; (iv) a reaction of 2-hydrazinyl-1,3,4-thiadiazine with ortho ester; (v) performing a Mannich reaction of 1,2,4-triazole-5-thiones, formaldehyde, and different primary amines; (vi) reacting *N*-(4*H*-1,2,4-triazol-3-yl) carboximidates with either carbon disulfide or sodium thiocyanate. This review provides a comprehensive investigation of the medical properties of triazolothiadiazine derivatives, focusing on their effects in areas such as cancer treatment, fighting against microbes, pain relief, reducing inflammation, antioxidant properties, antiviral effects, inhibiting enzymes, and combating tuberculosis.

## Abbreviations

p-TSOH	p-toluene-sulfonic acid
A549	Lung cancer cells
AcONa	Sodium acetate
DMF	Dimethyl formamide
DMSO	Dimethyl sulfoxide
EGFR	Estimated glomerular filtration rate
EtOH	Ethyl alcohol
G2/M phase	DNA damage checkpoint
HEK-293	Cell line from kidney cells
HEPG-2	Cell line from liver cancer
HIV-1	Human immunodeficiency virus-1
HIV-2	Human immunodeficiency virus-2
HPAI H5N1	A bird-adapted strain of H5N1 highly pathogenic avian Influenza A virus subtype H5N1
IC50	Half-maximal inhibitory concentration
i-PrOH	Isopropanol
MCF-12A	Breast cancer cell: Michigan Cancer Foundation-12A
MCF-7	Breast cancer cell line: Michigan Cancer Foundation-7
mg/kg/po	Milligram/kilogram by mouth
MT-4	Human T cell leukemia
MTT test	Dye of 2,5-diphenyl-2H-tetrazolium bromide assay to measure cell proliferation or cell cytotoxicity.
MWI	Microwave irradiation
DCM	dichloromethane
NBS	N-bromosuccinimide
PARP-1	Poly(ADP-ribose) polymerase-1
SNAr	Nucleophilic aromatic substitution reactions
T3P^®^	Propanephosphonic acid anhydride
TEA	Triethylamine
WST-1 test	Cell proliferation reagent
